# Deficiency of the Mycobacterial Lipoarabinomannan Biosynthesis Glycosyltransferase MptC Enhances Antibacterial Immune Response and Rifapicin Antibiotic Susceptibility

**DOI:** 10.3390/antibiotics15030291

**Published:** 2026-03-13

**Authors:** Jiaxin Hu, Hongliang Chen, Zhongkun Li, Hao Sun, Yi-Cheng Sun, Xiao-Lian Zhang

**Affiliations:** 1 State Key Laboratory of Virology and Biosafety, Hubei Province Key Laboratory of Allergy and Immunology, Department of Immunology, Wuhan University Taikang Medical School (School of Basic Medical Sciences), Wuhan University, Wuhan 430071, China2024203010042@whu.edu.cn (H.S.); 2 Frontier Science Center for Immunology and Metabolism, Department of Allergy, Zhongnan Hospital, Medical Research Institute, Wuhan University, Wuhan 430071, China; 3 NHC Key Laboratory of Systems Biology of Pathogens, National Institute of Pathogen Biology and Center for Tuberculosis Research, Chinese Academy of Medical Sciences & Peking Union Medical College, Beijing 100730, China

**Keywords:** *Mycobacterium smegmatis* (*M. smeg*), mannosyltransferase MptC, lipomannan, rifampicin, T cell cytokines production

## Abstract

Background/Objectives: The mycobacterial complex cell envelope serves as a formidable barrier against host immunity and antibiotics. Lipomannan (LM) and lipoarabinomannan (LAM) are key structural components of the mycobacterial envelope and potent immunomodulators. The mycobacterial lipoarabinomannan biosynthesis mannosyltransferase MptC modifies the multiple α-(1→2)-linked branched mannan residues of LAM in the mycobacteria. However, the role of MptC in mycobacterial infectivity, antibiotic susceptibility and host immune regulation remains poorly understood. Methods: An *mptC* (also named *MSMEG_4247*) knockout *Mycobacterium smegmatis* mc^2^-155 (*M. smeg*) strain (designated as *M. smeg*Δ*mptC*) was generated using CRISPR–Cas12a technology. The effects of MptC on bacterial physiology, cell wall permeability, drug sensitivity, immune cell function, and survival during infection are analyzed through glycogen staining, drug sensitivity tests, and cellular and mouse infection models. Results: MptC deficiency results in a loss of LM and increase in LAM synthesis. The *M. smeg*Δ*mptC* mutant strain exhibits enhanced cell wall permeability and reduces hydrophobicity. Functionally, the *mptC* knockout strain increases the intracellular cytokines (IFN-γ, TNF-a and IL-17) production of T cells in mice. Consequently, results based on both macrophage and mouse infection models demonstrate that the *M. smeg*Δ*mptC* strain has less bacterial loads and higher susceptibility to antibiotic rifampicin. Conclusions: Mannosyltransferase MptC plays an important role in maintaining cell wall integrity (via LM/LAM synthesis), regulating T cell responses, and influencing antibiotic susceptibility in mycobacteria.

## 1. Introduction

Tuberculosis (TB) is a chronic infectious disease caused by *Mycobacterium tuberculosis* (*M. tb*). It mainly affects the lungs but can also involve lymph nodes, bones, kidneys and other organs [1,2,3]. According to the 2025 report by the World Health Organization (WHO), in 2024, there were 10.7 million new cases of TB worldwide with approximately 1.23 million deaths [4]. The prevention and control of TB face significant challenges, particularly under the threat of drug-resistant TB, including multidrug-resistant TB (MDR-TB) and extensively drug-resistant TB (XDR-TB) [4]. Therefore, an in-depth exploration of the pathogenic mechanism of *M. tb*, as well as the development of new treatment strategies and vaccines, have become the current research focus.

The pathogenicity of *M. tb* is closely related to its unique cell wall structure [5]. The cell wall serves not only as a physical barrier for the bacteria but also participates in the regulation of the host immune system [6,7,8]. Lipoarabinomannan (LAM) is one of the main components of the cell wall of *M. tb*, consisting of a phosphatidylinositol core, mannose (Man) side chains and arabinose (Ara) side chains [9]. The structure of LAM is complex, and it can be classified into mannose-capped LAM (ManLAM), phospho-myo-inositol-capped LAM (PILAM) and non-capped LAM (Aral AM) based on the different terminal modifications [10,11]. The biosynthesis of LM/LAM involves the coordinated action of more than twenty enzymes [12]. During the synthesis process, the mannosyltransferase PimA is responsible for transferring the first mannose to the inositol moiety of phosphatidylinositol (PI), forming PIM1. Subsequently, the mannosyltransferase PimB (*Rv0557*, also termed MgtA) and PimB’ (*Rv2188c*) catalyze the transfer of the second mannose from GDP-mannose to PIM1, yielding PIM2 [13,14]. PIM2 is then acylated by PatA on one of its mannose residues and further mannosylated by PimC/PimD to generate the tetramannosylated glycolipid (AcPIM4). The downstream fate of AcPIM4 diverges: it may be mannosylated by theα-(1→2) mannosyltransferase PimE and another uncharacterized mannosyltransferase to form AcPIM6 [15], or AcPIM4 itself may serve as the substrate. Both AcPIM6 and AcPIM4 can be further mannosylated by α-(1→6) mannosyltransferases (e.g., MptB and MptA) [16] to generate lipomannan (LM). The long mannan backbone of LM can be further extended by the mannosyltransferase MptC, which adds multiple α-(1→2)-linked branched mannose residues [17,18]. Arabinosyltransferases (such as EmbC) can then attach the arabinan domain to the α-(1→6) mannan core to produce LAM.

Functional studies support the importance of this pathway in mycobacterial physiology and virulence. An *M. smegmatis* (*M. smeg*) *mptA* deletion mutant producing a small LM-precursor but no mature LM or LAM grew slowly on solid LB agar medium and failed to grow at 42 °C [19]. Knocking out the gene *pimB* involved in LM synthesis led to an increase in the mortality rate of the macrophages [20]. A key enzyme responsible for this backbone extension is MptC. This α-(1→2)-mannosyltransferase, encoded by *mptC* (also named named as *MSMEG_4247* in *M. smeg*; *Rv2181* in *M. tb* H37Rv), is crucial for generating the full-length mannan core branching. Using the *M. marinum*-zebrafish model, the mannan core branching of LM/LAM (not LAM capping) mediated by MptC is demonstrated to be critical for mycobacterial virulence in innate immunity [21]. The overexpression of *mptC* in *M. smeg* resulted in the truncation of the arabinan and mannan domains of LM and LAM, and similar changes in the apparent sizes of LM and LAM were observed in *M. tb* [22].

Despite accumulating evidence establishing MptC as a key regulator of LAM structural formation [18,23], the precise functions of MptC on bacterial physiology and host immune response have not been fully elucidated. In this paper, we aimed to investigate the functional role of MptC by generating an *mptC* knockout strain of *M. smeg*. Using CRISPR–Cas12a-mediated gene editing technology [24,25], we disrupted the *mptC* homolog (*MSMEG_4247*) and systematically examined the effects on the LAM structure, bacterial cell wall integrity, antibiotic susceptibility and host T cell immune responses. Our findings provide new insights into the roles of MptC-mediated LM/LAM biosynthesis in mycobacterial pathogenesis and host–pathogen interactions.

## 2. Results

### 2.1. MptC Knockout Does Not Affect the Expression of Its Upstream and Downstream Genes in M. smeg

**Figure 1:**
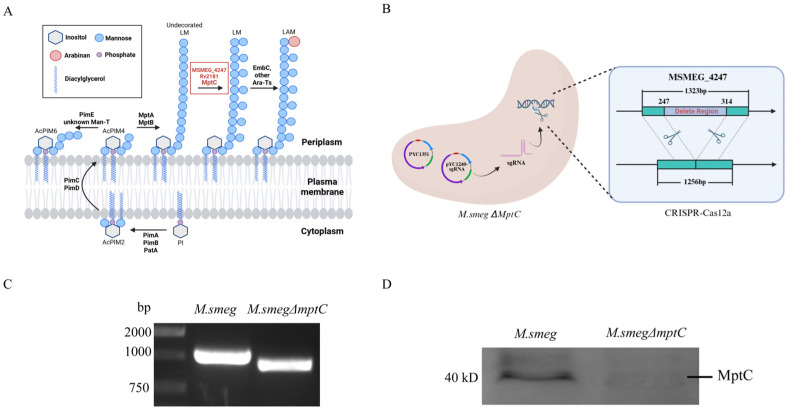
Verification of the *mptC (MSMEG_4247)* knockout strain and detection of the expression of related upstream and downstream genes. (**A**) Schematic illustration of the MptC function in the biosynthetic pathways of PIM, LM, and LAM. (**B**) CRISPR–Cas12a genome editing method for *M. smeg∆mptC.* (**C**) The *MSMEG_4247* gene in *M. smeg*∆*mptC* was partially deleted, resulting in a shorter gene length by PCR analysis. PCR product for *MSMEG_4247* of *M. smeg* = 1000 bp; PCR product for *MSMEG_4247* deletion of *M. smeg*∆*mptC* = 932 bp. (**D**) The successful knockout of *MSMEG_4247* in *M. smeg* was confirmed by Western blot analysis using a laboratory-generated rabbit polyclonal antibody. MptC molecular weight size is predicted as 48.2 kDa.

MptC modifies the multiple α-(1→2)-linked branched mannan residues of LAM in the mycobacterial LM/LAM biosynthetic pathway (Figure 1A). The *MSMEG_4247* gene was deleted in *M. smeg* using CRISPR–Cas12a genome editing (Figure 1B). The successful gene disruption in the *M. smeg*Δ4247 (referred to as *M. smeg*∆*mptC*) strain was confirmed by PCR (Figure 1C). Sequencing analysis reveals a 68 bp deletion (nucleotides 247–314) in the target gene (Figure S1A), resulting in both a frameshift mutation and premature termination.

To further verify the expression of *mptC* in *M. smeg* and *M. smeg*∆*mptC*, polyclonal antibodies against MptC were generated in New Zealand White rabbits. Western blot analysis confirms the successful knockout of mptC in *M. smeg*∆*mptC* (Figure 1D).

**Figure 2:**
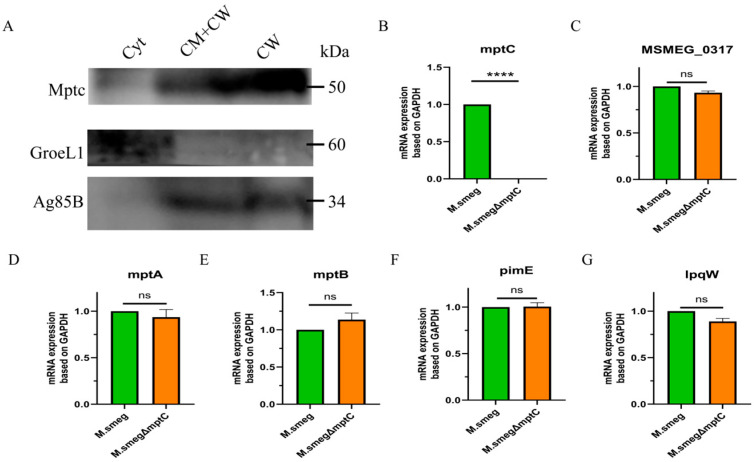
Verification of the *mptC (MSMEG_4247)* knockout strain and detection of the expression of related upstream and downstream genes. (**A**) Immunoblot analysis of subcellular fractions (Cyt, cytoplasm; CW, cell wall; CM, cell membrane) using anti-MptC antibody. The expected molecular weights are 48.2 kDa for MptC, 56.2 kDa for GroEL1 (cytosolic control), and 35 kDa for Ag5B (cell wall/membrane control). (**B**) The *MSMEG_4247* gene of *M. smeg*∆*mptC* was deleted successfully by RT-qPCR analysis. (**C**–**G**) The qRT-PCR validation of the transcriptional levels of the upstream and downstream genes of *mptC*. *n* = 3. Data are presented as mean ± SD from three independent experiments (*n* = 3). Statistical significance was assessed by unpaired two-tailed Student’s *t*-test. **** *p* < 0.0001; ns, not significant.

By analyzing separated fractions of the bacterial cell membrane, cell wall, and cytoplasm, we found that MptC is localized on the bacterial cell membrane and the cell wall (Figure 2A). Comparing the colony morphology of *M. smeg* and *M. smeg*∆*mptC* on solid media, both colonies exhibited a milky white color, and there was no significant difference in colony morphology observed with the naked eye (Figure S1B).

To detect the expression of the upstream and downstream genes of *mptC* in *M. smeg*∆*mptC*, bacterial RNA was extracted for RT-qPCR analysis. The results showed that the *mptC (MSMEG_4247)* gene was deleted successfully and *mptC* knockout did not affect the expression of its upstream (*mptA*, *MSMEG_0317*) or downstream (*mptB*, *pimE*, *lpqW*) genes (Figure 2B–G).

### 2.2. MptC Deficiency Affects the Production of Bacterial LM and LAM

**Figure 3:**
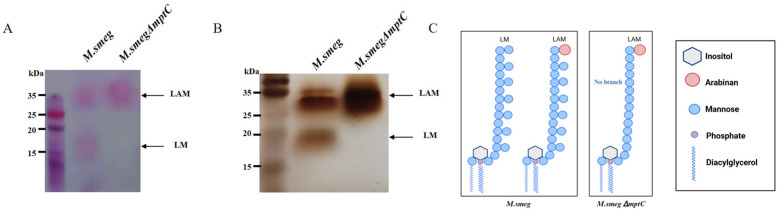
MptC deficiency affects the production of LM and LAM. (**A**) The purified LAM and LM components from *M. smeg* and *M. smeg*∆*mptC* were analyzed by glycogen staining (**A**) and silver staining (**B**). (**C**) Schematic diagram showing changes in LM/LAM between *M. smeg* and *M. smeg*∆*mptC*. This figure was modified from reference [23].

MptC is responsible for synthesizing the mannose side chains of LM and LAM. The knocking out of *mptC* is likely to affect the structure of LM and LAM on the bacterial surface. Since the genes for synthesizing LAM have a certain degree of functional redundancy, it is necessary to determine whether *mptC* gene knockout affected its product LAM/LM. Both *M. smeg* and *M. smeg*∆*mptC* cell wall lipomannan components were isolated and purified. Glycogen staining shows that the positions of LM and LAM in the wild-type *M. smeg* are 32 kDa and 18 kDa, respectively. Meanwhile, the LAM band became thicker, and the LM band disappeared in the *M. smeg*∆*mptC*, indicating an increase in LAM content and an absence in LM content (Figure 3A). Silver staining results display similar changes in the LAM structure (Figure 3B). Consistent with a previous report [23], these results can be attributed to the fact that LM lacking mannose side chains is susceptible to degradation unless it is further synthesized as LAM (Figure 3C).

### 2.3. MptC Knockout Increases Bacterial Cell Wall Permeability and Reduces Hydrophobicity

**Figure 4:**
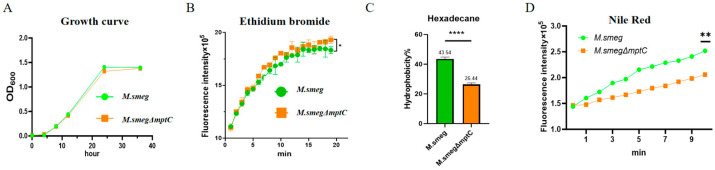
MptC deficiency compromises envelope integrity and alters cell surface properties in *M. smeg*. (**A**) Growth curves of *M. smeg* and *M. smeg*Δ*mptC* cultured in 7H9 medium. (**B**) EtBr uptake kinetics. (**C**) Cell-surface hydrophobicity assessed by the hexadecane partitioning assay and expressed as percentage adherence to hexadecane. (**D**) Nile Red uptake kinetics. Data are presented as mean ± SD from three independent experiments (*n* = 3). Statistical significance was analyzed by two-way ANOVA with Sidak’s multiple-comparison test for time-course data (**A**,**B**,**D**) and by a two-tailed unpaired Student’s *t*-test for panel (**C**). * *p* < 0.05, ** *p* < 0.01, **** *p* < 0.0001.

Next, we examined whether the changes in MptC affected the growth rate of bacteria. We detected the changes in the OD_600_ values of the bacterial solution at different time points. We found that the deletion of *mptC* did not significantly affect the growth rate of bacteria (Figure 4A). The ethidium bromide (EtBr) penetration experiment proved that the permeability of the bacterial cell wall increased after the deletion of *mptC* (Figure 4B). The hexadecane distribution experiment (Figure 4C) and the Nile red absorption experiment (Figure 4D) demonstrated that the hydrophobicity of the bacterial cell wall significantly decreased after the deficiency of *mptC*.

### 2.4. MptC Knockout Increases Bacterial Sensitivity to Rifampicin

**Figure 5:**
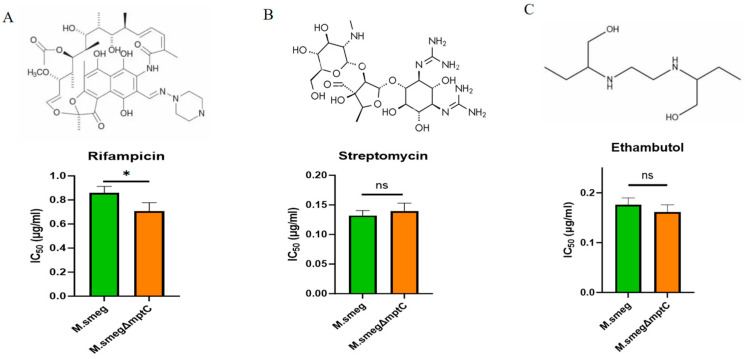
Detection of the effects of MptC deficiency on the susceptibility to antibiotics. (**A**–**C**) Upper panels with the chemical structures of rifampicin (**A**), streptomycin (**B**), and ethambutol (**C**). Lower panels show the corresponding IC_50_ values (µg/mL) for *M. smeg* and *M. smeg*Δ*mptC*. Data are presented as mean ± SD from three independent experiments (*n* = 3). Statistical significance was assessed using a two-tailed unpaired Student’s *t*-test (*M. smeg* vs. *M. smeg*Δ*mptC* for each antibiotic). * *p* < 0.05; ns, not significant.

The issue of drug resistance of *M. tb* is one of the main reasons for the difficulty in treating tuberculosis. Three kinds of *M. tb* antibiotics, rifampicin, streptomycin and ethambutol, with different structures as shown in Figure 5, were used to treat *M. smeg* and *M. smeg*∆*mptC*, respectively. We used gradient concentrations of rifampicin, streptomycin and ethambutol to stimulate *M. smeg* and *M. smeg*Δ*mptC*. After three days of treatment, we detected the bacterial cell-forming units (CFUs). After knocking out *mptC* in *M. smeg*, the sensitivity of *M. smeg* to rifampicin increased with a significantly lower IC_50_ (half-maximal inhibitory concentration) for rifampicin. This suggests that MptC may be involved in the process of *M. smeg* against rifampicin (Figure 5A). The increased sensitivity is possibly due to the enhanced permeability of the cell envelope to rifampicin upon *mptC* deletion, allowing more drug molecules to reach their intracellular target. This suggests that MptC contributes to *M. smeg* resistance against rifampicin, possibly by maintaining a cell envelope barrier that limits drug permeation. In contrast, the deletion of *mptC* did not significantly alter bacterial sensitivity to streptomycin or ethambutol (Figure 5B,C), which was possibly due to the different structures of drugs.

### 2.5. MptC Knockout Decreases Mycobacterial Invasion of Macrophages

**Figure 6:**
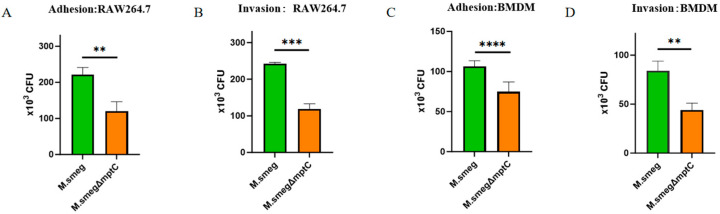
Deficiency of *mptC* reduces adhesion to and invasion of macrophages. (**A**,**B**) Adhesion (**A**) and invasion (**B**) of *M. smeg* and *M. smeg*Δ*mptC* in RAW264.7 macrophages. (**C**,**D**) Adhesion (**C**) and invasion (**D**) of *M. smeg* and *M. smeg*Δ*mptC* in primary BMDMs. Bacterial burdens were quantified as CFUs after infection as described in the Materials and Methods. Data are presented as mean ± SD from three independent experiments (*n* = 3). Statistical significance was assessed using a two-tailed unpaired Student’s *t*-test (*M. smeg* vs. *M. smeg*Δ*mptC* for each panel). ** *p* < 0.01, *** *p* < 0.001, **** *p* < 0.0001.

We performed the bacterial adhesion and invasion assays in macrophages at a multiplicity of infection (MOI) of 10. The results showed that *M. smeg* exhibited significantly greater adherence (2.22 × 10^5^ CFU of *M. smeg* vs. 1.20 × 10^5^ CFU of *M. smeg*Δ*mptC*, ** *p* < 0.01) and invasion (2.42 × 10^5^ CFU of *M. smeg* vs. 1.19 × 10^5^ CFU of *M. smeg*Δ*mptC*, *** *p* < 0.001) to RAW 264.7 macrophages (Figure 6A,B). Consistent results were observed in bacteria-infected mouse bone marrow–derived macrophages (BMDMs), where the *M. smeg*Δ*mptC* strain showed 29% reduced adhesion (1.06 × 10^5^ of *M. smeg* vs. 7.48 × 10^4^ CFU of *M. smeg*Δ*mptC*, **** *p* < 0.0001) and 47% lower invasion (8.40 × 10^4^ of *M. smeg* vs. 4.40 × 10^4^ CFU of *M. smeg*Δ*mptC*, ** *p* < 0.01) compared to wild-type (Figure 6C,D). These results suggest that MptC knockout impairs bacterial adhesion and the invasion of macrophages.

### 2.6. MptC Mediates Macrophages Binding Possibly via TLR2-TLR4 Complex

**Figure 7:**
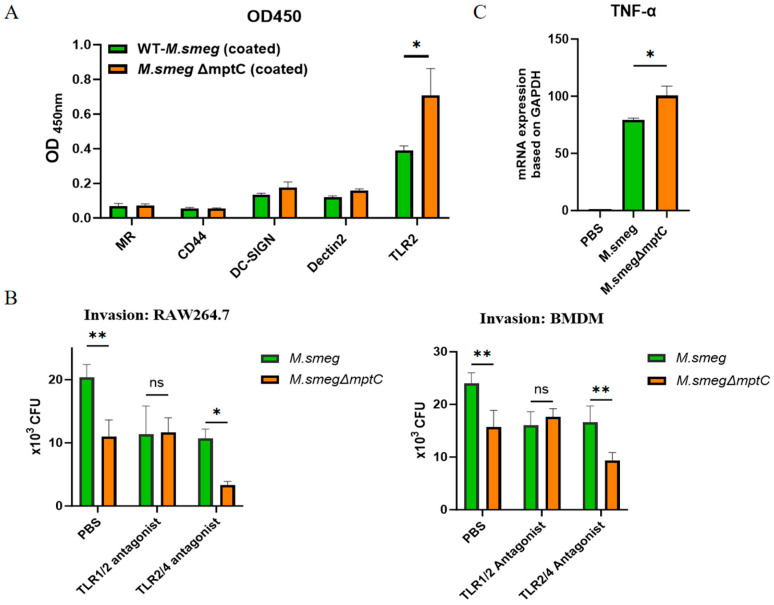
MptC contributes to TLR2/TLR4 complex-associated recognition and macrophage activation. (**A**) Binding signals (OD_450_) showing reduced TLR2 binding to *M. smeg*Δ*mptC*. (**B**) Macrophage invasion of RAW264.7 cells (left) and BMDMs (right) under the indicated treatments (PBS, TLR1/2 antagonist Cu-CRT22 (10 μM) or TLR2/4 antagonist Sparstolonin B (10 μM)), quantified as intracellular CFU. (**C**) TNF-α mRNA expression in RAW264.7 cells infected with *M. smeg* or *M. smeg*Δ*mptC* was assayed by RT-qPCR. Data are mean ± SD (*n* = 3). Statistics: one-way ANOVA with Tukey’s multiple-comparison test (**A**), two-way ANOVA (**B**) and two-tailed unpaired Student’s *t*-test (**C**). * *p* < 0.05, ** *p* < 0.01; ns, not significant.

*M. tb* can bind to various receptors on the surface of macrophage membranes. These receptors include complement receptors (CR1, CR3, or CR4), mannose receptors (MR), DC-SIGN, Dectin-2, Toll-like receptors (TLR) and NOD-like receptors. TLR2, CLRs MR, DC-SIGN, Dectin-2 and CD44 can recognize the glycolipid ManLAM on the surface of *M. tb* [26,27,28]. A ligand-binding enzyme-linked immunosorbent assay (ELISA) was performed to assess bacterial interactions with host receptors. *M. smeg* and the *M. smeg*Δ*mptC* mutant were separately immobilized on ELISA plates. The plates were then incubated with a panel of His-tagged recombinant host proteins, including MR, CD44, DC-SIGN, Dectin-2, and TLR2. After washing, bound proteins were detected using an HRP-conjugated anti-His antibody, which was followed by color development with a TMB substrate (Figure S2). ELISA experiments confirmed that the glycolipids on the surface of *M. smeg* mainly bound to the TLR2 receptor, and knocking out MptC further increased the binding ability of bacteria to the TLR2 receptor (Figure 7A).

Next, by treatment with the TLR1/2 antagonist Cu-CRT22 and TLR2/4 antagonist Sparstolonin B, we found that the TLR2/4 antagonist could reduce the invasion of *M. smeg* and *M. smeg*Δ*mptC* with a more pronounced effect on *M. smeg*Δ*mptC* (Figure 7B).

RT-qPCR analysis showed that TNF-α mRNA expression in *M. smegΔmptC-*infected RAW264.7 cells was significantly increased compared to that in *M. smeg* group (Figure 7C). Thus, the enhanced TLR2 engagement by *M. smeg*Δ*mptC* likely contributes to stronger macrophage activation with higher levels of TNF-α mRNA expression.

### 2.7. MptC Knockout Increases Intracellular IFN-γ, TNF-a and IL-17 Production in T Cells in Mice

**Figure 8:**
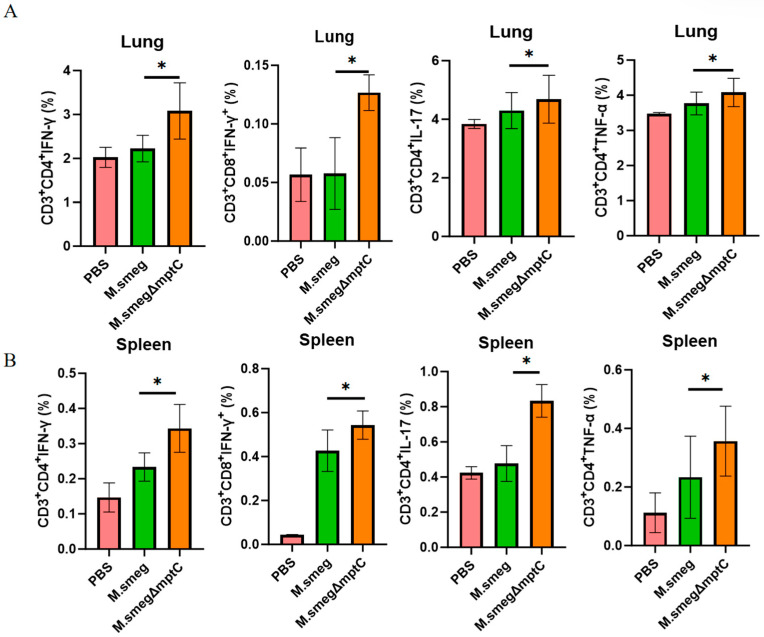
MptC deficiency enhances cytokine-producing T-cell responses during infection. Flow-cytometric quantification of intracellular cytokines (IFN-γ, TNF-α and IL-17) production in both CD4^+^ and CD8^+^ T cells of lung (**A**) and spleen (**B**) single-cell suspensions. Data are presented as mean ± SD (*n* = 3). Statistical significance was assessed by one-way ANOVA with Tukey’s multiple-comparison test. * *p* < 0.05.

Upon invasion, the mycobacterium is directly cleared by macrophages and also activates the adaptive immune response through antigen presentation. This response relies primarily on T lymphocyte-mediated cellular immunity to combat tuberculosis. To investigate the effect of MptC on T cell immune responses, *M. smeg* and *M. smeg*∆*mptC* were used to infect mice intranasally, and the spleen and lung T cell responses were detected at three days post-infection (Figure S3A). Mouse lung and spleen cell lysates were stimulated with heat-inactivated *M. smeg* (i*M. smeg*) followed by flow cytometry analysis. In the knockout group, IFN-γ production was significantly increased in both lung and splenic CD8^+^ and CD4^+^ T cells (Figure 8A,B). While both lung and splenic CD4^+^ T cells also showed elevated IL-17 and TNF-α production (Figure 8A,B). IFN-γ and TNF-α can act on infected target cells to eliminate and kill mycobacteria. IL-17 is involved in protective immunity against tuberculosis [29]. These results suggest that *mptC* knockout promotes the host’s T cell cytokine response to *M. smeg*, aiding in the clearance of bacteria.

### 2.8. MptC Knockout Reduces M. smeg Loading in Mouse Lungs and Enhances Rifapicin Antibiotic Susceptibility

**Figure 9:**
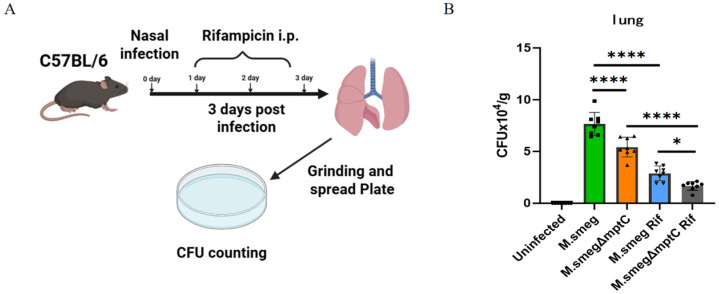
MptC knockout reduces *M. smeg* survival in mouse lungs and enhances rifapicin antibiotic susceptibility. (**A**) Experimental timeline. Mice were infected intranasally with *M. smeg* or *M. smeg*Δ*mptC* with or without rifampicin (Rif) treatment. Bacterial loads in lungs were assessed 3 days post-infection. (**B**) Lung CFU counts. Data are mean ± SD (*n* = 8 per group; * *p* < 0.05, **** *p* < 0.0001, by Welch and Brown–Forsythe test). Rif: rifampicin.

Next, we further examined the effect of *mptC* knockout on the bacterial loads and antibiotic resistance to rifampicin in a mouse infection model. *M. smeg* and *M. smeg*Δ*mptC* were used to infect mice intranasally (i.n.) with daily rifampicin administration by intraperitoneal injection (i.p.) (Figure 9A). After three days post-infection, the mouse lungs were ground in PBS and diluted to prepare the dilution mixture for spreading on plates. The single colonies growing on the plates for three days were counted. We observed that after knocking out *mptC*, the bacterial load of *M. smeg* in the mouse lungs significantly decreased (Figure 9B). Furthermore, MptC deficiency led to an increase in bacterial susceptibility to rifampicin (Figure 9B). In the mouse model, rifampicin treatment achieved a 14% higher bacterial inhibition rate in the *M. smeg*Δ*mptC* group compared to the *M. smeg* group. This might be due to the absence of *mptC* leading to an increase in both antibacterial immune response and rifapicin antibiotic susceptibility.

## 3. Discussion

Tuberculosis caused by *M. tuberculosis* is a top killer among infectious diseases. Despite the approval of several new antibiotics, including bedaquiline, delamanid, linezolid, and pretomanid, more *M. tuberculosis* strains resistant to these drugs have emerged. Therefore, continuous improvement and new targets against TB are urgently required. *M. tuberculosis* involves a complex pathogenic mechanism involving intricate interactions between the bacterium and the host immune system. The mycobacterial mannosyltransferase MptC is a key enzyme for modifying the mannan core branching of LAM in the mycobacterial wall. Until now, the role of MptC in mycobacterial characteristics, antibiotic susceptibility and host immune regulation remains poorly understood. In this paper, we demonstrate that MptC-mediated mannosylation plays an important role in maintaining the proper LAM structure and mycobacterial virulence. *M. smeg*∆*mptC* strain has less bacterial infectivity and higher susceptibility to antibiotic rifampicin in both macrophages and mice. Functionally, the MptC knockout strain increases the pro-inflammatory cytokines production of T cells in mice. To our knowledge, this is the first report showing that MptC deficiency increases the pro-inflammatory cytokines production of T cells in mouse infection models.

In this paper, we demonstrate that MptC deficiency leads to the disappearance of LM and an increase in LAM content. This result aligns with the reported function of MptC [23]—namely, its role in synthesizing the mannose side chain of LAM, whose expression directly influences LAM’s structural integrity. Alterations in the LAM structure further affect bacterial physiological traits: *mptC* deletion increases cell wall permeability and decreases hydrophilicity. Notably, changes in *mptC* expression significantly affect bacterial drug sensitivity: *mptC* deletion enhances bacterial sensitivity to rifampicin but not to streptomycin or ethambutol. Rifampicin targets bacterial RNA polymerase. Its penetration into mycobacteria largely depends on passive diffusion through the hydrophobic mycolic acid layer due to its apolar structure (characterized by a naphthol ring and an ansa bridge). Rifampicin is particularly sensitive to alterations in the cell wall permeability. The deletion of *mptC*, by disrupting the lipoarabinomannan layer and the outer membrane integrity, likely facilitates this diffusion process, thereby specifically enhancing rifampicin’s access to its intracellular target. The susceptibility to ethambutol remains not significantly changed, as its molecular target (arabinosyltransferases in arabinan synthesis) is distinct from the functional role of MptC in mannan chain extension. The persistent streptomycin susceptibility likely stems from its hydrophilic nature, which favors entry through porin channels—a pathway largely independent of the hydrophobic barrier altered by *mptC* deletion. This suggests that MptC-dependent LAM modifications act as a natural barrier against certain antibiotics, such as rifampicin.

Interestingly, our findings appear to contrast with a study on an *M. marinum mptC* mutant [21]. In that study, the mutant’s antibiotic susceptibility profile (including to streptomycin, erythromycin, isoniazid, rifampicin, polymyxin B, and chloramphenicol) was reported to be similar to that of the wild-type *M. marinum*, whereas our *M. smeg*Δ*mptC* showed increased sensitivity to rifampicin. This discrepancy might be attributed to species-specific differences or the method of mutant construction. The *M. marinum* mutant was generated by the transposon insertion of 286 nucleotides downstream of the start codon of *mmar_3225* (an orthologue of *M. tb Rv2181*), which resulted in the production of smaller LAM and LM molecules. However, in another study on *M. smeg*, the absence of *mptC* led to the abolition of LM synthesis, which is consistent with our results. The absence or/and presence of LM in different species indicate that these macromolecules have complex regulation in different species, and further research is needed to explain this phenomenon.

We have confirmed that the MptC-regulated lipid-associated membrane (LAM) structure is crucial for the interaction between bacteria and host immune cells. Knockout strains exhibit significantly impaired adhesion and invasion abilities toward macrophages, and it also led to increased production of pro-inflammatory cytokines (TNF-α, IFN-γ, and IL-17) by T cells. Here, the bacterial survival in mouse infection experiments demonstrated that the *M. smeg*∆*mptC* mutant strain exhibited significantly lower lung CFU and stronger immune response compared to the *M. smeg* strain, indicating impaired survival and colonization capability upon *mptC* deletion in vivo. This finding is consistent with previous observations, where an *mptC* mutant also showed markedly attenuated virulence [21].

Members of the TLR family, including TLR2, TLR4, and TLR9, are involved in the immune response to *M. tb* infection. TLR2 recognizes surface lipoproteins and/or lipoglycans, promoting the maturation of antigen-presenting cells and the secretion of TNF-α, IL12, and IFN-γ. TLR4 recognizes the lipopolysaccharide (LPS), and TLR9 recognizes the non-methylated CpG DNA of *M. tb* [30]. Notably, although *M. smeg*∆*mptC* showed enhanced TLR2 binding, it exhibited reduced invasion or internalization into macrophages (Figure 6). This result might be due to the distinct roles of TLRs for *M. smeg*∆*mptC*: TLR2/4 might primarily function as a signaling receptor that promotes macrophage activation, pro-inflammatory cytokine production and immune response rather than acts as a phagocytic receptor. LAM may also induce autophagy-related processes by TLR2 and activate the expression of pro-inflammatory cytokines [31,32]. The response of B cells to LAM was shown to occur in a TLR2-dependent manner [33]. The impact of TLR2 on bacterial adhesion, invasion, and signaling pathways remains to be determined using TLR2 inhibitors or TLR2 knockout mice. Further resolving LAM–receptor complex structures via surface plasmon resonance (SPR) or cryo-electron microscopy would help to illuminate the molecular basis of their immunomodulatory effects in future studies.

Despite revealing the significant role of MptC in LAM synthesis and host immune regulation, this paper has several limitations. First, *M. smeg*, a non-pathogenic bacterium, might have a distinct LM/LAM composition and host interactions compared to virulent *M. tb*; future studies should verify MptC’s function in virulent *M. tb*. Second, although the transcription levels of the *mptC* neighboring genes were unchanged, genetic complementation of the *M. smeg*∆*mptC* mutant strain would further strengthen the causal attribution of the observed phenotypes. Third, the receptor-binding ELISA and pharmacological inhibition suggest the involvement of TLR2/TLR4-associated recognition, but genetic receptor knockout/knockdown or biophysical binding assays would be needed. Finally, the in vivo experiments were focused on an early (3-day) time point and a limited antibiotic pane; extended infection time courses and broader drug testing may further delineate the role of MptC in persistence and drug tolerance.

In summary, this paper reveals for the first time the crucial role of MptC in mediating mycobacterial tolerance to rifampicin and the pro-inflammatory cytokines production of T cells. This paper also provides a new perspective for mycobacterial cell wall biology and its interaction with host immune cells. Targeting cell wall components like MptC may thus represent a potential strategy for developing adjunctive therapies against mycobacterial infections.

## 4. Materials and Methods

### 4.1. Construction of MptC Knockout Strain of M. smeg and Observation of Bacterial Morphology

*M. smeg* strains [34] used in this paper were cultured on Middlebrook 7H10 agar medium (Becton, Dickinson and Company (BD) (Franklin Lakes, NJ, USA)) supplemented with 10% (*v*/*v*) OADC (oleic acid–albumin–dextrose–catalase) and 0.5% (*v*/*v*) glycerol. The plates were streaked with bacterial stocks and incubated at 37 °C under aerobic conditions for 48–72 h until single colonies appeared.

The *mptC* gene was disrupted in *M. smeg* via CRISPR–Cas12a-mediated genome editing basically according to previous publications [25,35]. The shuttle plasmid pYC1240 (pCR-hyg) was used to express sgRNA [24]. The helper plasmid pYC1351 (pNHEJ-Cas12a-recAmu) carries mycobacterial NHEJ-dependent *IigD*, *KuC*, and *NrgA* genes for increasing NHEJ repair efficiency [24]. First, the shuttle plasmid pYC1240 was digested with *Bpm*I and *Hin*dIII, and the linearized backbone was gel-purified. The sgRNA sequence used in this paper is 5′-gctcgccgcgatcgcattcgctccg-3′, which targets nucleotides 258–282 of the *mptC* gene (*MSMEG_4247*). sgRNAs primers were designed to flank the MptC target region and ligated into the digested pYC1240. The pYC1240-sgRNA construct was then electroporated into the competent *M. smeg* harboring the helper plasmid pYC1351 and then plated on Middlebrook 7H10 agar supplemented with 10% OADC and 0.5% glycerol along with kanamycin (25 μg/mL), hygromycin (50 μg/mL), and anhydrous tetracycline (100 ng/mL), which was followed by incubation at 37 °C for 4 days. The *M. smeg*∆*mptC* mutant was confirmed by genomic DNA PCR analysis. *M. smeg* genomic DNA was used as a control. Primers targeting the region flanking the sgRNA were designed as follows: forward primer: 5′-ctggagatgagtaagcggcag-3′; Reverse primer: 5′-acaccggtgagaccaccaggc-3′. The expected amplicon sizes were 1000 bp for *M. smeg* and 932 bp for the *M. smeg*Δ*mptC* mutant strain. PCR products were purified and subjected to Sanger sequencing to confirm the correct gene disruption. *M. smeg* and the MptC knockout strain of *M. smeg* were applied onto the 7H10 agar containing hygromycin, respectively, and the morphological differences were observed for 3 days.

### 4.2. Purification and Identification of LAM/LM from M. smeg

The LAM extraction procedure was performed basically as previously described [36]. Bacterial cultures were grown to mid-log phase (OD_600_ = 0.6–0.8), harvested by centrifugation, and washed three times with PBS. The pellet was lyophilized overnight, which was followed by sequential delipidation: (1) first extraction with chloroform:methanol (2:1, vol/vol) at a 1:9 (wt/vol) ratio at 37 °C with shaking overnight and (2) second extraction with chloroform:methanol:water (10:10:3, vol/vol/vol) under the same conditions. The delipidated pellet was air-dried in a fume hood, resuspended in PBS, and subjected to enzymatic lysis using lysozyme (20 mg/mL), PMSF (1:1000 dilution), and DNase I (100 U/mL) at 37 °C for 2 h, which was followed by sonication. The lysate was mixed with 8% Triton X-114 (vol/vol), incubated at 4 °C overnight, and phase-separated at 37 °C. The aqueous phase was combined with 9 volumes of 95% ethanol, precipitated at −80 °C overnight, and the pellet was air-dried. Finally, the material was digested with proteinase K (0.24 mg/mL) at 60 °C for 2 h, and the supernatant was analyzed by glycostaining.

For visualization by silver staining, equal amounts of the purified LAM/LM preparations were resolved by SDS–PAGE and stained using a standard silver-staining procedure. Gels were fixed in 40% (*v*/*v*) ethanol and 10% (*v*/*v*) acetic acid, rinsed with water, sensitized with 0.02% (*w*/*v*) sodium thiosulfate, and incubated in 0.1% (*w*/*v*) silver nitrate. Bands were developed in 2% (*w*/*v*) sodium carbonate containing 0.04% (*v*/*v*) formaldehyde, and the reaction was stopped with 5% (*v*/*v*) acetic acid prior to imaging.

For glycogen detection, the gel was fixed and oxidized in a solution containing 30% ethanol, 10% acetic acid, and 0.7% periodic acid for 10 min with agitation. After washing with deionized water (3 × 3 min), it was stained with Schiff’s reagent for 20 min in the dark. Finally, the gel was destained with multiple washes in deionized water until clear bands were visualized.

### 4.3. Reverse Transcription-Quantitative Real Time PCR (RT-qPCR)

Total RNA was extracted from wild-type and knockout bacterial strains at mid-log phase (OD_600_ 0.6–0.8) using the RNeasy Mini Kit (Qiagen, Singapore, Cat. #74104) with modifications: 7 mL of bacterial culture was centrifuged, washed twice with EDTA-Tris buffer (pH 8.0), and subjected to three freeze–thaw cycles (−80 °C). Cell pellets were resuspended in RLT buffer, vortexed vigorously, and lysed by sonication. Lysates were centrifuged (12,000× *g*, 5 min), and supernatants were mixed with absolute ethanol before loading onto spin columns. After sequential washes with RW1 buffer (twice) and RPE buffer (once), RNA was eluted in nuclease-free water and reverse-transcribed using a SYBR Green Real-Time pCR Master Mix plus RT-pCR (Biotechnology Overseas (Osaka, Japan)).

### 4.4. Bacterial Physiological Characterization and Cell Wall Permeability

All experiments were performed using bacteria in the logarithmic growth phase. To assess bacterial growth kinetics, optical density measurements of bacterial culture were taken at 2 h intervals from an initial culture (~2 × 10^7^ CFU/mL, OD_600_ = 0.02). For ethidium bromide (EtBr) uptake assay, bacterial cells were resuspended in PBS supplemented with glucose, which was followed by the addition of EtBr at a final concentration of 1 μg/mL. Fluorescence intensity was monitored using an excitation wavelength of 530 nm and an emission wavelength of 590 nm [37].

Similarly, Nile red uptake assay was evaluated by resuspending bacterial cells in glucose-supplemented PBS and incubating with 10 μM Nile red. Fluorescence was measured at an excitation wavelength of 540 nm and an emission wavelength of 600 nm [30].

Bacterial hydrophobicity was determined using the hexadecane adhesion assay. Briefly, bacterial suspensions in PBS were mixed with hexadecane at a ratio of 5:4 (*v*/*v*, bacterial suspension: hexadecane). After vigorous mixing and phase separation, the aqueous phase was collected, and the OD_600_ values were measured to determine cell adhesion [38].

### 4.5. Antimicrobial Resistance Assay

To assess drug resistance, wild-type and knockout bacterial strains were cultured to the logarithmic growth phase (OD_600_ =0.6–0.8). Bacterial suspensions were adjusted to an initial OD_600_ of 0.02 and treated with serially diluted concentrations of the target antibiotic for 72 h. Bacterial growth was monitored by measuring OD_600_, and the IC_50_ was calculated using GraphPad Prism.

### 4.6. Bacterial Adhesion and Invasion Assays in Macrophages

BMDMs were isolated from the femurs and tibias of C57BL/6 mice. Mice were euthanized by an intraperitoneal injection of sodium pentobarbital, and hind limbs were aseptically removed. Bone marrow cavities were flushed with ice-cold RPMI 1640 medium (containing 10% FBS and 1% penicillin–streptomycin) to obtain bone marrow cells. After centrifugation at 600× *g* for 5 min at 4 °C, cells were treated with ACK lysis buffer for 5 min at room temperature to remove red blood cells. Lysis was stopped by adding excess RPMI 1640 medium, which was followed by two washes with the same medium. Cells were resuspended in DMEM supplemented with 10% FBS, 1% penicillin–streptomycin, and 40 ng/mL recombinant murine M-CSF, and then they were seeded into 12-well plates at 1 × 10^6^ cells/well. Cells were cultured at 37 °C with 5% CO_2_. After three days incubation, non-adherent cells were removed, and fresh DMEM containing 40 ng/mL M-CSF was added. BMDMs were obtained for experiments on day 6–7 upon reaching confluence.

Mouse Raw264.7 macrophages or BMDMs were seeded in a 12-well plate, at a density of 1 × 10^6^ cells per well, and cultured in 1 mL of 1640 cell culture medium for 20 h prior to infection. The cells were infected at an MOI of 10. For adhesion assay, after 1 h of infection, cells were washed twice with PBS to remove non-adherent bacteria. Macrophages were lysed with 0.2% Triton X-100 for 5 min, and serial dilutions of the lysates were plated to quantify adherent bacterial CFUs. For invasion assay, following 1 h of infection, extracellular bacteria were removed by PBS washing. Cells were incubated for 1 h in gentamicin-containing medium to kill remaining extracellular bacteria. The medium was replaced with fresh antibiotic-free medium, and macrophages were further incubated for 4 h to allow intracellular bacterial replication. Cells were lysed with 0.2% Triton X-100, and serial dilutions were plated to determine invaded bacterial CFUs.

### 4.7. ELISA-Based Ligand-Binding Assay

Wells of a 96-well plate were coated with 100 µL of 0.1 mg/mL poly-L-lysine (Merck KGaA, Darmstadt, Germany) and incubated at 4 °C for 1 h. Following this, either M. smeg or *M. smeg*∆*mptC* was then immobilized onto the coated wells. After blocking, recombinant His-tagged pattern recognition receptors (PRRs), including MR, CD44, DC-SIGN, Dectin-2, and TLR2 (IPODXI, Wuhan, China), were added to the wells and incubated at 37 °C for 2 h. The wells were then washed to remove unbound proteins. Bound His-tagged receptors were detected by incubation with an HRP-conjugated anti-His antibody (ABclonal, Wuhan, China) at 37 °C for 1 h. Finally, the reaction was developed with TMB substrate, stopped with sulfuric acid, and the absorbance was measured at 450 nm.

### 4.8. Mouse Infection and Lung CFU Enumeration

C57BL/6 mice (6–8 weeks old) were used for infection experiments. C57BL/6 mice were selected for this paper due to their documented use in tuberculosis research and their association with a Th1-skewed response, which is protective against intracellular pathogens [39,40]. Mice were lightly anesthetized with isoflurane and intranasally inoculated with WT *M. smeg* or *M. smeg*∆*mptC* at a dose of 1 × 10^8^ CFU in 50 µL PBS. Control mice received PBS only. Where indicated, rifampicin was administered by intraperitoneal injection according to the experimental design (dose and schedule described elsewhere/see figure).

At 3 days post-infection, mice were euthanized and lungs were aseptically collected and weighed. Lung tissues were homogenized in sterile PBS, and homogenates were serially diluted and plated on Middlebrook 7H10 agar for CFU determination. Plates were incubated at 37 °C for 4 days before colony counting. Bacterial burden is reported as CFU per gram of lung tissue (CFU/g). The inhibition rate was calculated as follows: [1 − (mean CFU of treated group/mean CFU of untreated control)] × 100%.

Group sizes (for lung CFU counting: *n* = 8 per group) were chosen based on sample sizes commonly used in comparable murine mycobacterial infection to minimize animal use in accordance with the 3R (Replacement, Reduction, and Refinement) principles [41].

All animal experiments were conducted in accordance with international and national guidelines for the care and use of laboratory animals. This paper was approved by the Institutional Animal Care and Use Committee (IACUC) of Wuhan University (Approval No.: WP20240336; Date of approval: 5 July 2024).

Specific-pathogen-free (SPF) female C57BL/6 mice (aged 6–8 weeks) were purchased from Skobees Biotechnology Co., Ltd. (Anyang, Henan, China). All mice were healthy and without genetic modifications. At the end of each experiment, euthanasia was performed on the mice by an intraperitoneal injection of sodium pentobarbital, and every effort was made to minimize their suffering.

### 4.9. Flow Cytometry Detection of T Cell Cytokines Production in a Mouse Infection Model

Groups of mice were inoculated intranasally with 10^8^ CFU of either *M. smeg* or *M. smeg*∆*mptC* mutant in a volume of 30 µL PBS; control mice received PBS only. Three days post-infection, mice were euthanized. Spleen and lung organs were aseptically harvested and prepared into single-cell suspensions. For intracellular cytokine staining, cells were restimulated ex vivo with heat-inactive *M. smeg* (i*M. smeg*) at an MOI of 10 for 6 h in the presence of GolgiPlug (protein transport inhibitor). Following stimulation, cells were stained for surface markers (CD3, CD4, CD8), fixed, permeabilized, and then stained intracellularly for cytokines production in T cells (IFN-γ, TNF-α, IL-17). Data were acquired on a flow cytometer and analyzed to determine the frequency of cytokine-positive cells within CD4^+^ and CD8^+^ T cell populations.

Group sizes (for flow cytometry analysis: *n* = 3 per group) were chosen based on sample sizes commonly used in immunophenotyping studies and to minimize animal use in accordance with the 3R principles [41].

### 4.10. Statistical Analysis

Data were analyzed using GraphPad Prism (version 9.0.2). Normality was assessed using the Shapiro–Wilk test, and the homogeneity of variances was assessed using the Brown–Forsythe/Levene test (for ANOVA) or F test (for *t* test). For two-group comparisons, an unpaired two-tailed Student’s *t*-test was used when assumptions were met (for data with normal distribution but unequal variances, Welch’s *t* test was used); otherwise, the Mann–Whitney U test was applied. For comparisons among more than two groups, one-way ANOVA with Tukey’s multiple-comparison test was used when assumptions were met (for data with normal distribution but unequal variances, Welch and Brown–Forsythe tests were used); otherwise, the Kruskal–Wallis test with Dunn’s post hoc test was applied. For time-course or two-factor designs, two-way ANOVA with appropriate multiple-comparison correction was used when assumptions were met (for data with normal distribution but unequal variances, Geisser–Greenhouse correction was used). Statistical significance was set at *p* < 0.05. Asterisks in the figures indicate the following levels of significance: * *p* < 0.05; ** *p* < 0.01; *** *p* < 0.001; **** *p* < 0.0001.

## 5. Conclusions

In summary, MptC deletion alters LAM structure, thereby affecting bacterial cell wall traits, drug sensitivity, and interactions with host immune cells. These findings deepen our understanding of mycobacterial infection and immune mechanisms, and they provide a theoretical basis for developing novel anti-TB drugs or vaccines. Future research should further clarify the precise relationship between LAM structure and immune regulation to drive innovative progress in TB prevention and treatment.

While our findings highlight the roles for MptC in LM/LAM homeostasis, envelope properties, host immune responses and rifampicin susceptibility, the conclusions are currently based on a non-pathogenic *M. smeg* model and an early infection time point. Future work should validate these observations in pathogenic mycobacteria (e.g., *M. tb*), including genetic complementation, and structural and genetic approaches to define the receptor pathways involved, as well as evaluating a broader range of antibiotics and longer infection courses.

## Data Availability

The data supporting the findings of this study are available within the article and its Supplementary Materials. Raw data (e.g., flow cytometry FCS files and CFU count spreadsheets) are available from the corresponding authors upon reasonable request.

## Supplementary Materials

The following supporting information can be downloaded at https://www.mdpi.com/article/10.3390/antibiotics15030291/s1, Figure S1: Bacterial sequencing results and colony morphology. (A) sanger sequencing of the PCR product confirmed the deletion of nucleotides 247 to 314 in the *M. smeg*∆*mptC* (B) Morphological observation of *M. smeg* and *M. smeg*∆*mptC* strain; Figure S2. Schematic of the receptor-binding ELISA. Plates were coated with *M. smeg* or *M. smeg*∆*mptC* and incubated with recombinant His-tagged receptors (MR, CD44, DC-SIGN, Dectin-2, and TLR2); binding was detected with an HRP–anti-His antibody and read at OD450; Figure S3. MptC deficiency enhances cytokineproducing T-cell responses during infection. (A) Experimental design schematic. Mice were intranasally inoculated with PBS, *M. smeg*, or *M. smeg*∆*mptC* (1 × 10^8^ CFU) and analyzed 3 days postinfection. (B,C) Gating strategy for intracellular cytokine staining.The spleen (B) and lung (C) gating strategy for intracellular cytokine staining is shown in the figure.The cytokine circle gate location was determined by FMO contol.
