# Supplementary material for: Deficiency of the Mycobacterial Lipoarabinomannan Biosynthesis Glycosyltransferase MptC Enhances Antibacterial Immune Response and Rifapicin Antibiotic Susceptibility
Source: Antibiotics (Basel). 2026 Mar 13;15(3):291. doi: 10.3390/antibiotics15030291 (PMC13023972; doi:10.3390/antibiotics15030291)
Supplement: Supplementary file 1 [file antibiotics-15-00291-s001.zip › antibiotics-4153506-supplementary.pdf]

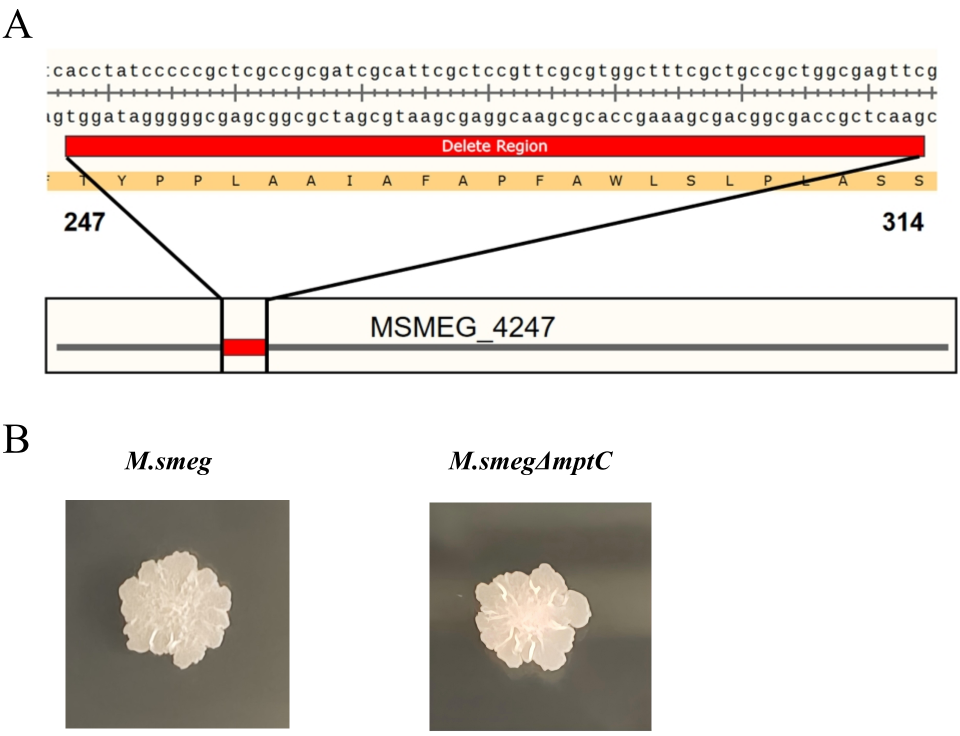

Supplement Figure S1. Bacterial sequencing results and colony morp hology. (A)sanger sequencing of the PCR product confirmed the delet ion of nucleotides 247 to 314 in the *M. smegΔMptC*(B)Morphological observation of *M.smeg* and *M.smegΔmptC* strain.

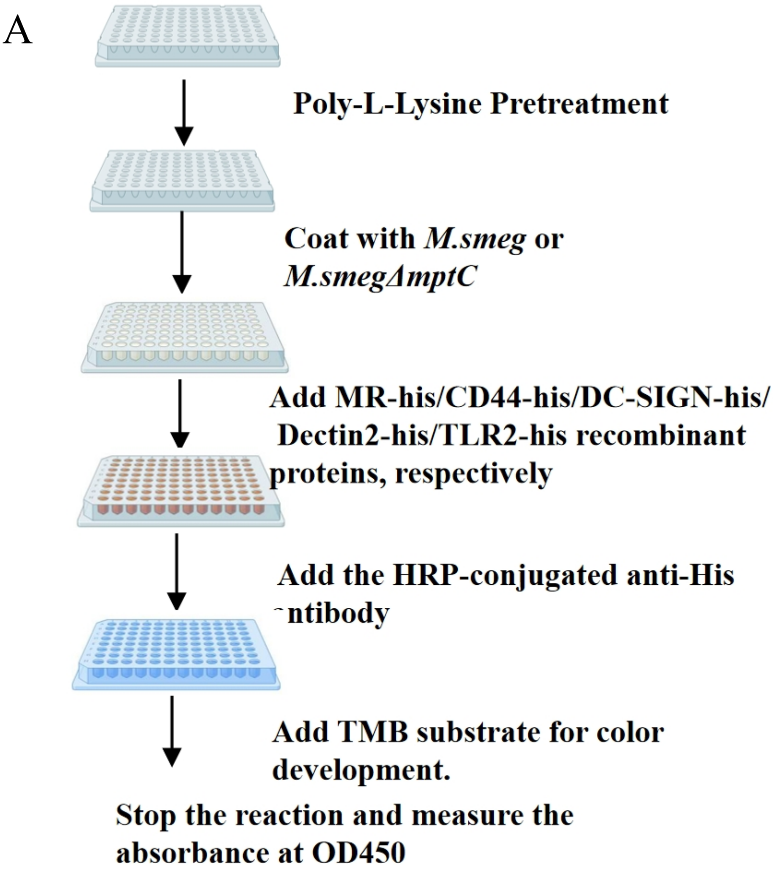

Supplement Figure S2. Schematic of the receptor-binding ELISA. Plates were coated with *M.smeg* or *M.smegΔmptC* and incubated with recombinant His-tagged receptors (MR, CD44, DC-SIGN, Dectin-2, and TLR2); binding was detected with an HRP－anti-His antibody and read at OD450.

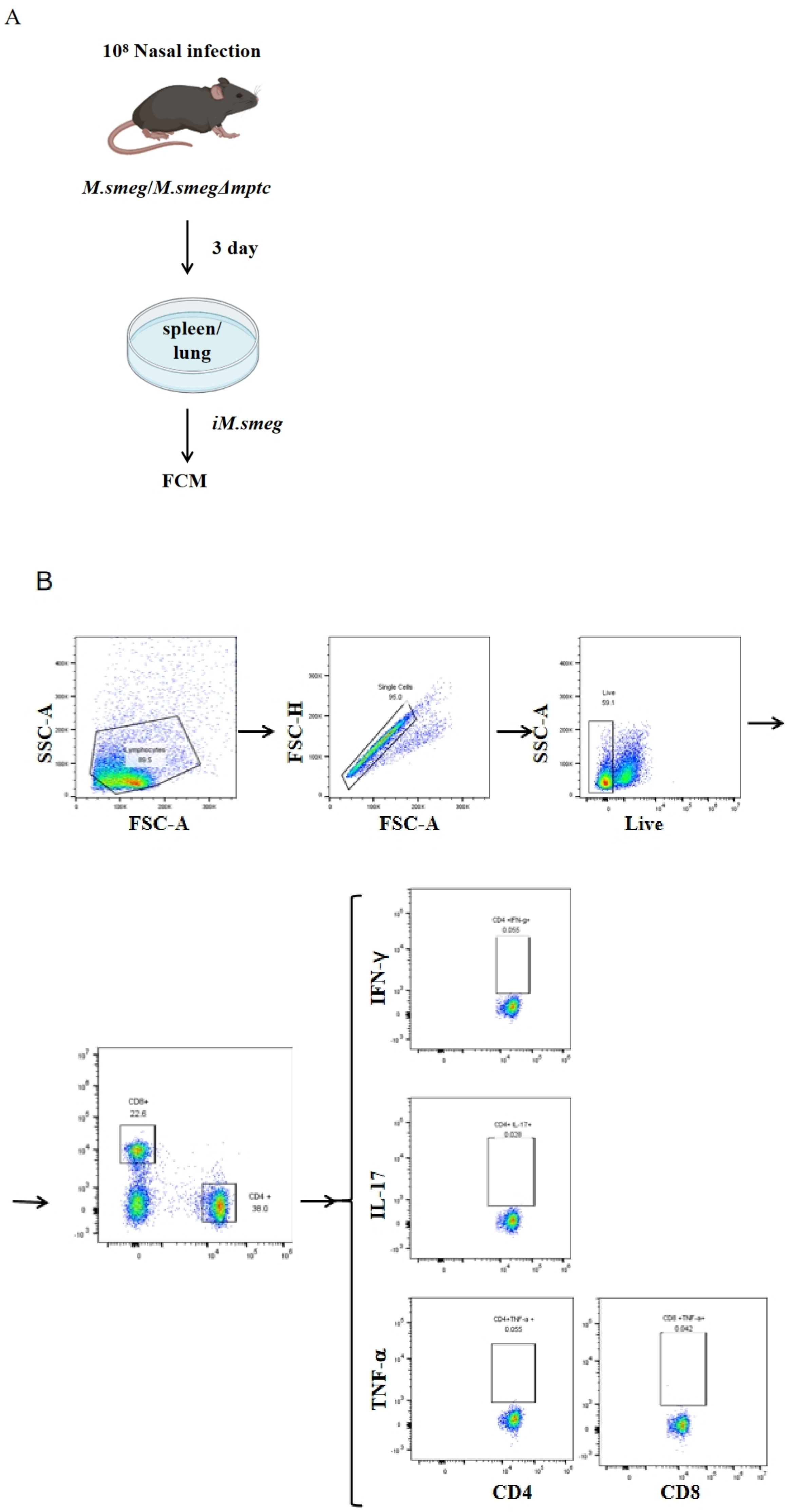
A
10⁸ Nasal infection
M.smeg/M.smegΔmptc
3 day
spleen/
lung
iM.smeg
FCM
B
SSC-A
FSC-A
FSC-H
FSC-A
SSC-A
Live
CD8+
22.6
CD4 +
38.0
IFN-γ
IL-17
TNF-α
CD4
CD8

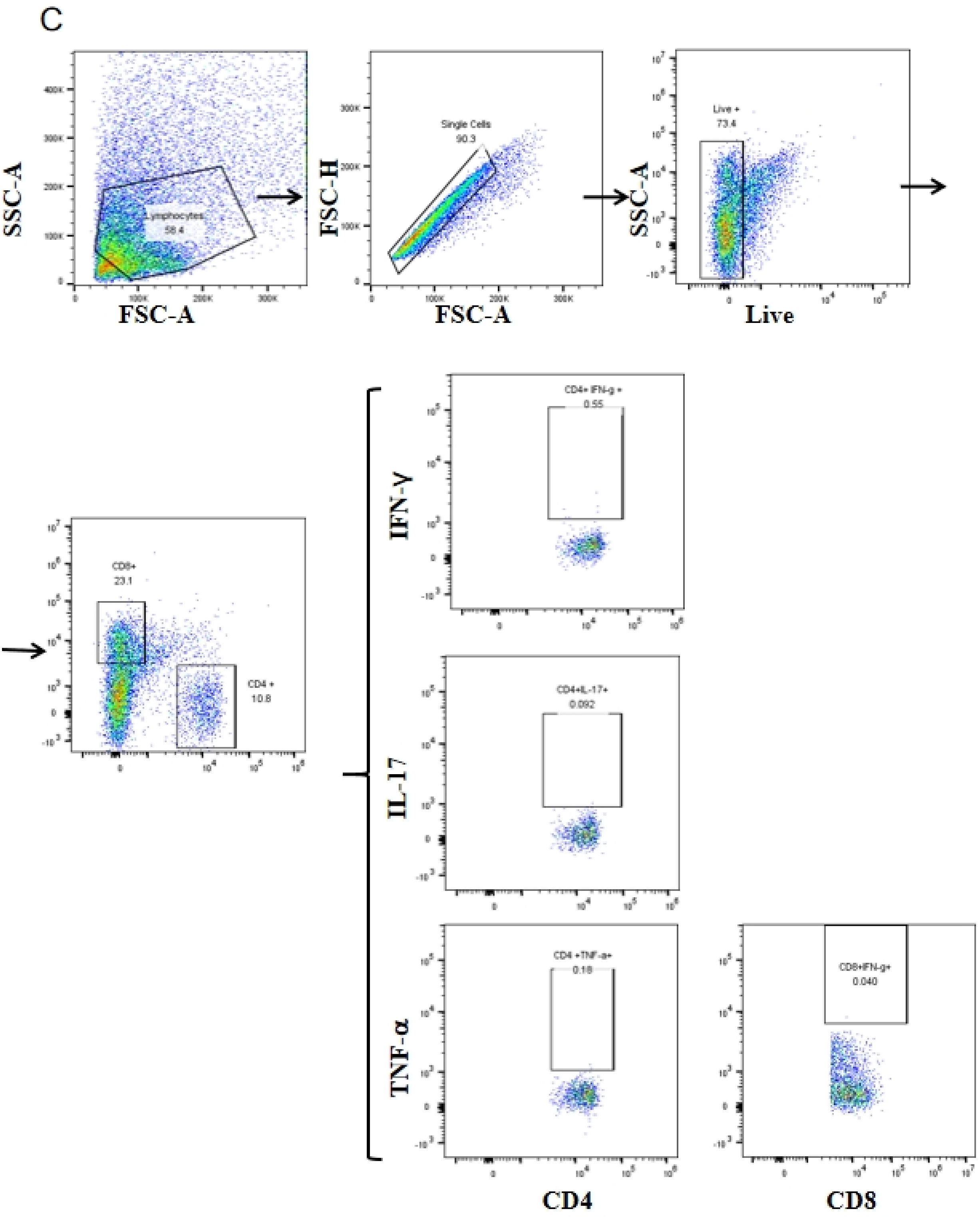

Supplement Figure S3. MptC deficiency enhances cytokine-producing T-cell responses during infection. (A) Experimental design schematic. Mice were intranasally inoculated with PBS, *M. smeg*, or *M. smegΔmptC* (1×10^8 CFU) and analyzed 3 days post-infection.(B,C)Gating strategy for intracellular cytokine staining.The spleen (B)and lung(C)gating strategy for intracellular cytokine staining is shown in the figure.The cytokine circle gate location was determined by FMO contol.
